# Risk assessment by client and case manager for shared decision making in outpatient forensic psychiatry

**DOI:** 10.1186/s12888-015-0500-3

**Published:** 2015-05-27

**Authors:** Rob H. S. van den Brink, Nadine A. C. Troquete, Harry Beintema, Tamara Mulder, Titus W. D. P. van Os, Robert A. Schoevers, Durk Wiersma

**Affiliations:** Department of Psychiatry, University of Groningen, University Medical Center Groningen, CC73, PO Box 30.001, 9700 RB Groningen, The Netherlands; Mental Health Organisation Lentis and Forensic Psychiatric Clinic Dr. S. van Mesdag, Groningen, The Netherlands; Mental Health Organisation Drenthe, Assen, The Netherlands; Mental Health Organisation Friesland, Leeuwarden, The Netherlands

**Keywords:** Client perspective, Self-assessment, Violence risk assessment, Shared decision making, Care planning, Outpatient forensic psychiatry

## Abstract

**Background:**

In outpatient forensic psychiatry, assessment of re-offending risk and treatment needs by case managers may be hampered by an incomplete view of client functioning. The client’s appreciation of his own problem behaviour is not systematically used for these purposes. The current study tests whether using a new client self-appraisal risk assessment instrument, based on the Short Term Assessment of Risk and Treatability (START), improves the assessment of re-offending risk and can support shared decision making in care planning.

**Methods:**

In a sample of 201 outpatient forensic psychiatric clients, feasibility of client risk assessment, concordance with clinician assessment, and predictive validity of both assessments for violent or criminal behaviour were studied.

**Results:**

Almost all clients (98 %) were able to fill in the instrument. Agreement between client and case manager on the key risk and protective factors of the client was poor (mean kappa for selection as key factor was 0.15 and 0.09, respectively, and mean correlation on scoring −0.18 and 0.20). The optimal prediction model for violent or criminal behaviour consisted of the case manager’s structured professional risk estimate for violence in combination with the client’s self-appraisal on key risk and protective factors (AUC = 0.70; 95%CI: 0.60–0.80).

**Conclusions:**

In outpatient forensic psychiatry, self-assessment of risk by the client is feasible and improves the prediction of re-offending. Clients and their case managers differ in their appraisal of key risk and protective factors. These differences should be addressed in shared care planning. The new Client Self-Appraisal based on START (CSA) risk assessment instrument can be a useful tool to facilitate such shared care planning in forensic psychiatry.

**Electronic supplementary material:**

The online version of this article (doi:10.1186/s12888-015-0500-3) contains supplementary material, which is available to authorized users.

## Background

Clinicians providing compulsory treatment, as in forensic psychiatry, have a ‘dual role’ [1]. They work in the interest of both the client and the community. Prominent for the latter is the need to be protected from violent or criminal behaviour. Apart from the medical model, forensic psychiatry is dominated by two treatment models which originated from the correctional setting [2]. The Risk-Need-Responsivity (RNR) model emphasizes that treatment should focus on changeable factors which have been shown to be related to the client’s risk for re-offending; i.e. his ‘criminogenic needs’ [3]. Based upon an assessment of these needs, the clinician selects the appropriate treatment targets. In this last respect, the RNR model resembles the medical model. Marshall and Bibby refer to this as ‘the belief that the professional knows what is best for the client’ [4]. The Good Lives Model (GLM), on the other hand, stresses that re-offending is best prevented by helping the client build a ‘good’-that is a personally fulfilling-life, and develop socially acceptable ways to satisfy his basic human needs [5]. Here, treatment targets are selected upon what the client values as sources of subjective well-being in his life. Neither of these models, however, appears to strike the right balance between the competing interests of the client and the community, which is at the heart of the dual role of forensic psychiatric clinicians.

Shared Decision Making (SDM) has been suggested as an intermediate approach between the paternalistic attitude of the medical and RNR models and the ‘client decides’ approach conveyed by the GLM model [6]. Essential features of SDM are an exchange of information, covering both the professional and personal perspective on the problem, and a commitment to build consensus on treatment targets. SDM has been widely propagated in medicine [7–10], and has been shown to increase client satisfaction, treatment adherence and quality of life in longer lasting treatment relations [11, 12]. Of special interest for forensic psychiatry is SDM’s explicit aim to get clients more involved in their treatment [13, 14]. It has been noted, however, that offering clients some degree of autonomous choice in their treatment planning may be particularly challenging in situations where that treatment is involuntary [8, 15, 16].

In the current study we test an instrument that could be used to facilitate SDM in routine forensic psychiatric care. Numerous risk assessment instruments have been developed [17], which recently may also include protective factors [18]. These instruments are used to assess re-offending risk and to identify the treatment needs of the client [19]. However, none of these instruments is designed to elicit the client’s view on treatment needs. In an earlier study we found that client-clinician contact in outpatient forensic psychiatry may be too brief, infrequent, and setting specific for clinicians to acquire the comprehensive view of their clients’ life and functioning necessary for adequate risk assessment [20]. Therefore, we developed an instrument to elicit the client’s perspective on his treatment needs, based on an established risk assessment tool; the Short Term Assessment of Risk and Treatability (START) [21]. The START addresses risk and protective factors, and is suitable for monitoring client functioning in inpatient and outpatient settings.

The current study aims to investigate the feasibility of administering our new ‘Client Self-Appraisal based on START’ instrument, the concordance between client and clinician risk assessments, and the predictive validity of both assessments for incidents of violent or criminal behaviour. Together this will show the opportunities for, and potential of, the client instrument to contribute to shared care planning in outpatient forensic psychiatry.

## Methods

### Client self-appraisal based on START

The Client Self-Appraisal based on START (CSA; see Additional file 1) was developed as part of an intervention studied in a randomized controlled trial on risk assessment and shared care planning in outpatient forensic psychiatry in the Netherlands (Study acronym RACE: Risk Assessment and Care Evaluation; trial registration NTR1042) [22]. Administration of the client instrument was the first step in a structured approach to shared care planning, and was meant to have clients assess their own primary risk and protective factors for poor functioning and re-offending.

Our objective was to develop a questionnaire that is easy to understand for many clients, and therefore is in simple wording, without double negations. It consists of reformulations of the 20 vulnerability and strength factors of the START (listed in Table 1), which should capture the essence of the original items and be acceptable for clients as self-descriptions. Initial discussions with clinicians led to the addition of one new item, on sexuality, which they considered indispensable for many of their forensic psychiatric outpatients. In the analyses on correspondence between the CSA and START in the present study, this additional item was not used, because there is no corresponding item on the START. As in the START, clients have the opportunity to add additional risk and protective factors, if they feel these are not covered by the items provided.

**Table 1:** Frequencies of selection of item as key factor by clients and case managers (*n* = 194^*^)

START item	Critical vulnerability						Key strength					
	Clients			Case managers			Clients			Case managers		
	Frequency	Percent	Rank	Frequency	Percent	Rank	Frequency	Percent	Rank	Frequency	Percent	Rank
Social skills	29	14.9	11	20	10.3	13	30	15.5	8.5	25	12.9	11
Relationships	37	19.1	6.5	51	26.3	4	30	15.5	8.5	31	16.0	7
Occupational	37	19.1	6.5	30	15.5	6.5	52	26.8	1	67	34.5	1
Recreational	20	10.3	13	23	11.9	11.5	42	21.6	3.5	40	20.6	4.5
Self-Care	14	7.2	15	9	4.6	17.5	15	7.7	17.5	28	14.4	9
Mental state	36	18.6	8	29	14.9	8.5	22	11.3	15.5	8	4.1	20
Emotional state	47	24.2	2	60	30.9	2.5	29	14.9	10	17	8.8	13.5
Substance use	32	16.5	9	36	18.6	5	28	14.4	11	42	21.6	3
Impulse control	49	25.3	1	60	30.9	2.5	32	16.5	6	16	8.2	15
External triggers	21	10.8	12	23	11.9	11.5	22	11.3	15.5	9	4.6	19
Social support	30	15.5	10	29	14.9	8.5	45	23.2	2	58	29.9	2
Material resources	44	22.7	4	17	8.8	14	42	21.6	3.5	32	16.5	6
Attitudes	5	2.6	19	25	12.9	10	23	11.9	14	17	8.8	13.5
Medication adherence	7	3.6	17	16	8.2	15	15	7.7	17.5	26	13.4	10
Rule adherence	9	4.6	16	8	4.1	19.5	8	4.1	20	22	11.3	12
Conduct	3	1.5	20	9	4.6	17.5	11	5.7	19	12	6.2	17
Insight	38	19.6	5	30	15.5	6.5	31	16.0	7	30	15.5	8
Plans	15	7.7	14	14	7.2	16	24	12.4	12.5	15	7.7	16
Coping	46	23.7	3	70	36.1	1	37	19.1	5	11	5.7	18
Treatability	6	3.1	18	8	4.1	19.5	24	12.4	12.5	40	20.6	4.5

*For 2 of the 196 first assessments by a client there was no START by the case manager

First, clients are asked about their risk factors for poor functioning and re-offending. Risk factors are called ‘vulnerability points’, and are defined in the questionnaire as ‘things in yourself or in your life which can put you at risk for not doing well, and can bring you into contact with the police and legal system again’. Clients indicate for each item whether or not it is a risk factor for them (answering options: yes, somewhat, no). Then they mark their 3 or more most important (‘key’) risk factors. This procedure is repeated for the client’s ‘protective points’. Contrary to the original START, this first part of the questionnaire does not yet assess how well the client is currently doing on these factors. Therefore, a second part was added, in which clients rate how well they are doing at the moment on their self-selected key factors, using an 11-point rating scale, ranging from 0 (‘could not be worse’) to 10 (‘could not be better’). Separate mean scores are calculated for the key risk and key protective factors, which are designated-in accordance with the START terminology-as ‘Client mean critical vulnerabilities’ and ‘Client mean key strengths’ score, respectively.

### Study setting and participants

The RACE-study was conducted between September 2007 and September 2010 in three outpatient forensic psychiatric services in the north of the Netherlands [22]. Eligible were all case managers and clients of the participating services. We defined case managers as those with primary responsibility for the care planning of their clients. Excluded were clients with expected discharge within 6 months or with less than one treatment contact per month. To avoid spill over of treatment effect, case managers with their whole caseload were randomized to Intervention or Care-As-Usual. Clients had a follow-up period of 18 months, or until end of care or end of study, if this occurred earlier. All clients lived in the community during this period. Informed consent was asked for client interviews. Approval was obtained from the Dutch Medical Ethical Committee for Mental Healthcare.

The present study concerns the clients and case managers randomized to the intervention arm of the RACE-study. The intervention consisted of a structured approach to risk assessment and shared care planning, to be implemented at all evaluations of the client’s treatment plan, but at least once a year. In preparation of the treatment evaluation, the case manager assessed the client’s risk and protective factors for re-offending with the START. Independently, the client did the same, using Part 1 of the CSA. In this way, both the case manager and client identified the client’s key risk and protective factors, to be addressed in the new treatment plan. In the subsequent treatment evaluation session, the case manager and client discussed these factors in a structured way. This discussion was aimed at agreement on the targets and content of the new treatment plan. As part of the structured procedure in the treatment evaluation session, clients also rated their current functioning on the self-selected key risk and protective factors, using Part 2 of the CSA. Case managers had been trained in the use of the START and the structured approach to shared care planning, described more fully elsewhere [22]. Clients received no training in the use of the CSA, but case managers were prepared to deal with any questions which might arise.

### Case manager risk assessment on the START

Case managers in the intervention arm of the RACE-study assessed their clients on the START [21]. The START is a risk assessment instrument consisting of 20 items scored both as ‘vulnerabilities’ (i.e. risk factors) and ‘strengths’ (protective factors). There is room for the addition of two client specific items. First, current client functioning is rated by scoring each item as ‘minimally present’ (0), ‘moderately present’ (1) or ‘maximally present’ (2). Next, items perceived by the case manager as of particular importance for the client are identified and marked as, respectively, ‘critical vulnerabilities’ and ‘key strengths’. In accordance with the structured professional judgment approach to risk assessment [23], final risk estimates are then asked for 7 outcomes, by weighing the identified vulnerabilities and strengths and applying clinical judgment. Clients are scored as being at ‘low’ (1), ‘medium’ (2) or ‘high’ (3) risk for these outcomes. The final risk estimate for ‘violence against others’ covers the primary outcome of the present study best, and was included in the prediction analyses below. Furthermore, apart from the usual START sum scores of all vulnerabilities and strengths, also the mean scores on the selected critical and key items were calculated, in order to obtain case manager rated predictors which are comparable to those obtained from risk assessment by the client.

### Incidents of violent or criminal behaviour

The primary outcome of the RACE-study consisted of incidents of violent or criminal behaviour by the client during a follow-up period. In the present study the predictive validity of client and case manager assessments of risk and protective factors for such incidents in the six months following assessment are compared. In the RACE-study prediction of incidents over a six month period proved to be better for case manager assessments on the START than over the regular three months period, probably due to a too low base rate of incidents for shorter periods [20, 21]. Violent behaviour is defined as any intentional behaviour with the potential to physically harm a person or animal or any seriously threatening or intimidating aggression. Criminal behaviour additionally covers exhibitionism, possession of child pornography, stalking, drug dealing, driving without a license or under influence, possession of an illegal weapon, vandalism, and theft. Not included is the use of illegal drugs, since this is not considered a crime under Dutch law.

Case managers recorded incidents that could potentially satisfy the above definitions on a standard form in the client’s case file. Inclusion as an incident of violent or criminal behaviour was determined through consensus between three experts in outpatient forensic psychiatry, who were blind to the risk assessments by the client and case manager. Outcome consisted of either absence or presence of one or more incidents during the six months following the treatment evaluation session, in preparation of which both the client and case manager conducted their risk assessment.

### Analysis

The feasibility of the Client Self-Appraisal based on START was assessed by the number of clients who were able to fill in the instrument at least once and the number of missing answers in these assessments.

Concordance between client and case manager risk assessment was studied in three ways. First, on a group level, frequencies by which items were chosen as key factor by case managers and clients were compared and tested with a Spearman rank correlation. Second, on an individual level, agreement between client and case manager on the items chosen as key factors for that particular client were tested with Cohen’s kappa corrected for agreement by chance, and averaged over the risk and protective factors separately. Third, also on the individual level, the mean correlation was calculated between client and case manager scoring of current client functioning on the key factors selected by the client.

The predictive validity of client and case manager risk assessment for incidents of violent or criminal behaviour in the next six months was studied by logistic regression analysis and the Area Under the Curve (AUC) statistic of Receiver Operating Characteristic (ROC) analysis [24]. Client assessments only covered the factors they selected as critical vulnerability or key strength. Case manager assessments in addition included the sum scores of all 20 vulnerability and strength items of the START, and the case manager’s final risk estimate for violence against others. Both univariate and multivariate analyses were conducted. In the multivariate analysis all client and case manager predictors were examined together, to reach an optimal prediction model for incidents of violent or criminal behaviour, using stepwise backward elimination of predictors based on the likelihood ratio test.

## Results

### Participants

**Fig. 1:**
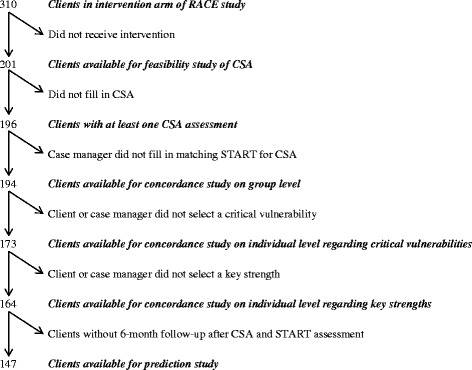
Flow chart of numbers of clients available for subsequent analyses

Nineteen case managers and 310 clients were included in the experimental arm of the RACE-study. A detailed description of their characteristics is provided elsewhere [22]. Of the 310 clients, 201 (65 %) actually received the intervention that included the CSA (see flow chart in Fig. 1). These 201 clients were predominantly male (93 %), 39.9 years old on average (s.d. = 10.9), and had been in outpatient forensic care for a mean period of 23.2 months (s.d. = 22.7) before inclusion in the RACE-study. They had a history of (a combination of) violent offences (59 %), sexual offences (35 %), property offences (38 %), or other offences (23 %), and were in treatment because of a criminal or civil treatment order (19 %), compulsory probation supervision (26 %), or voluntarily (56 %). On Axis I they were diagnosed with an impulse control disorder (26 %), paraphilia (21 %), psychotic disorder (8 %), substance related disorder (42 %), other mental disorder (55 %), or no disorder (6 %). In addition, 72 % had a personality disorder, mainly of the Cluster B type (26 %), or Not Otherwise Specified (35 %), and 10 % had borderline intellectual functioning or less. Their mean score on the historical risk factors of the HCR-20 risk assessment instrument [25], which assesses criminally relevant history, was 7.9 (s.d. = 3.8). On none of these characteristics the clients who received an intervention differed significantly from those who were randomized to the experimental arm of the study but did not receive the intervention (all *p* > 0.05).

Different numbers of clients were available for the subsequent analyses of the present study (see Fig. 1). Reasons for drop-out or exclusion at the different stages are specified below.

### Feasibility of risk assessment by the client

Of the 201 clients who received an intervention, 196 clients (98 %) filled in the CSA at least once. In total 297 interventions were conducted, for which clients filled in the client instrument 282 times (95 %) and case managers the START 293 times (99 %). The CSA was completed once by 132 clients (67 %), twice by 44 clients (22 %), three times by 18 clients (9 %), and four times by two clients (1 %).

In the 196 first assessments on the CSA per client, the average percentage of missing answers (i.e. clients who did not provide a valid answer) on the risk factors was 0.7 % (range 0–2.6 % over the different factors), and 2.0 % (1.0–4.6 %) on the protective factors. For comparison, the average percentages of missing answers in the corresponding STARTs by the case manager were: 0.4 % (0–2.6 %) and 0.5 % (0–2.6 %), respectively. Selection of key risks was missing in 8.2 % of the first client assessments, of key strengths in 10.7 %, and of both in 5.6 %. In the corresponding case manager STARTs these percentages were 3.5, 5.6, and 3.0 %, respectively.

### Concordance on group level

Table 1 presents the rank ordered frequencies by which clients and case managers selected the START items as critical vulnerability or key strength of the client on their first assessment per client. The Spearman rank correlation between the distributions for clients and case managers was 0.81 (95 % CI: 0.58–0.92) for the critical vulnerabilities and 0.51 (95 % CI: 0.10–0.77) for the key strengths. This good concordance between clients and case managers on a group level is illustrated by the fact that both groups choose the same 3 factors most frequently as critical vulnerability (namely ‘Emotional Sate’, ‘Impulse Control’, and ‘Coping’) and that 2 of the 3 most frequently chosen key strengths corresponded (‘Occupational’ and ‘Social Support’).

### Concordance on individual level

Table 2 shows the agreement between individual clients and their case manager on the selection of START items as a critical vulnerability or key strength of that client. The level of agreement was assessed by Cohen’s kappa. A value of 1 indicates perfect agreement, −1 perfect disagreement, and 0 an agreement that is no better than chance. Only first CSA assessments per client and their matching START assessment by the case manager were included, with as additional selection criterion that both the client and case manager had selected at least one critical vulnerability (leaving *n* = 173 pairs of assessments), respectively one key strength (*n* = 164).

**Table 2:** Agreement between client and case manager on selection of key factors for client and rating of client functioning

START item	Critical vulnerability (*n* = 173)		Key strength (*n* = 164)	
	Kappa for selection	Correlation for functioning	Kappa for selection	Correlation for functioning
Social skills	0.12	−0.08	0.04	0.24
Relationships	0.33^*^	0.06	−0.06	0.23
Occupational	0.29^*^	−0.31	0.22^*^	0.32^*^
Recreational	0.10	−0.53^*^	0.14	0.48^*^
Self-Care	0.21^*^	0.33	0.21^*^	0.39
Mental state	0.08	0.03	0.07	−0.13
Emotional state	0.04	−0.26	0.18^*^	−0.07
Substance use	0.36^*^	−0.09	0.02	0.29
Impulse control	0.27^*^	−0.14	−0.03	0.18
External triggers	0.24^*^	−0.55^*^	0.00	0.40
Social support	0.19^*^	−0.43^*^	0.17^*^	0.22
Material resources	0.12	−0.37^*^	0.11	0.36^*^
Attitudes	0.03	−0.41	0.10	−0.13
Medication adherence	0.24^*^	−0.83	0.27^*^	−0.48
Rule adherence	0.21^*^	0.30	0.08	−0.15
Conduct	−0.03	−0.25	0.03	0.89^*^
Insight	0.00	0.07	0.10	0.60^*^
Plans	0.07	−0.34	0.05	−0.04
Coping	0.09	−0.26	0.01	0.39
Treatability	0.12	0.46	0.08	0.00

*p < 0.05

The mean kappa across the START items for agreement on selection of critical vulnerabilities was 0.15 (95%CI: 0.11–0.20), which is limited. This is also shown by the fact that if the case manager selected an item as critical vulnerability of the client, the mean chance across the items that the client also selected that item as critical vulnerability was only 28 % (95%CI: 24–32 %). For the selection of key strengths, the mean kappa was 0.09 (95%CI: 0.05–0.13), and the mean chance of the client selecting the same item as key strength as his case manager was 24 % (95%CI: 20–28 %).

Table 2 also shows the correlation between client and case manager scoring of current client functioning on the items selected by the client as critical vulnerability or key strength. As described above, a high client score (on 0–10 scale) indicates good client functioning, for both vulnerabilities and strengths. A high case manager score (on 0–2 scale), on the other hand, indicates good functioning for strengths, but poor functioning for vulnerabilities.

A mean correlation across the vulnerabilities of −0.18 (95 % CI: −0.10 to −0.26) was found, and of 0.20 (0.12 to 0.28) across the strengths. Both show limited agreement between the client and case manager on client functioning, in the expected direction. The unweighted mean score by clients across the critical vulnerabilities they selected was 5.75 (95%CI: 5.57–5.93), which indicates that they saw their functioning on these factors as almost sufficient. On the strengths the clients scored 7.32 (7.18–7.46) on average, which denotes good functioning according to the client. The case managers were somewhat less positive about client functioning. Their corresponding scores were 0.90 (0.84–0.97) on average for the vulnerabilities and 1.31 (1.25–1.36) for the strengths, indicating a moderate presence of vulnerabilities and clear presence of strengths.

### Predictive validity of client and case manager risk assessments

Available were 175 pairs of risk assessments by both client and case manager, concerning 147 clients, with non-overlapping six month follow-up periods (range 1–3 per client). In 34 (19 %) of the follow-up periods at least one incident of violent or criminal behaviour occurred (see [22] for a specification of types of incidents).

The univariate relationships between client and case manager risk assessments and the occurrence of incidents of violent or criminal behaviour are presented in the top half of Table 3. Both client self-assessments on critical vulnerabilities and key strengths were significant univariate predictors, as were the sum scores and final risk estimate of the case manager. However, the vulnerabilities and strengths selected by the case managers as of particular importance for the client, were unrelated to re-offending by that client. As noted above, contrary to the case manager assessments, higher scores on client assessment of vulnerabilities indicate better functioning. The observed univariate relationships in Table 3 are therefore all in the expected direction of a decreased risk with better client functioning. The predictive powers of the separate predictors were ‘modest’ [26], with a maximum AUC of 0.65 for the client assessment of key strengths as the best individual predictor of client violent or criminal behaviour.

**Table 3:** Predictive validity of client and case manager risk assessments for violent or criminal behaviour (*n* = 175)

Predictor	B (s.e.)	p	OR (95 % CI)	AUC (95 % CI)	p
*Univariate analyses*					
Client mean critical vulnerabilities	−0.26 (0.13)	0.037	0.77 (0.60–0.98)	0.62 (0.52–0.72)	0.032
Client mean key strengths	−0.33 (0.14)	0.019	0.72 (0.54–0.95)	0.65 (0.55–0.74)	0.009
Case manager mean critical vulnerabilities	0.20 (0.40)	0.617	1.22 (0.56–2.67)	0.53 (0.42–0.64)	0.609
Case manager mean key strengths	−0.52 (0.49)	0.289	0.60 (0.23–1.55)	0.54 (0.43–0.65)	0.471
Case manager sum vulnerabilities	0.08 (0.03)	0.008	1.08 (1.02–1.14)	0.63 (0.52–0.74)	0.018
Case manager sum strengths	−0.05 (0.03)	0.080	0.95 (0.90–1.01)	0.61 (0.52–0.71)	0.043
Case manager risk estimate for violence	0.79 (0.33)	0.015	2.21 (1.16–4.18)	0.62 (0.51–0.73)	0.030
*Multivariate analysis*					
Client mean critical vulnerabilities	−0.28 (0.13)	0.040	0.76 (0.59–0.99)		
Client mean key strengths	−0.28 (0.14)	0.048	0.75 (0.57–1.00)		
Case manager risk estimate for violence	0.77 (0.34)	0.024	2.15 (1.11–4.18)		
				0.70 (0.60–0.80)	<0.001

The optimal multivariate prediction model is presented in the bottom half of Table 3. This model includes both the case manager’s final risk estimate for violence against others and the client’s assessments on critical vulnerabilities and key strengths. These factors had mutually independent contributions to the prediction of violent or criminal behaviour by the client in the following six months. Their combined predictive power was ‘acceptable’ [26], with an AUC of 0.70, which corresponds to a medium Cohen’s effect size *d* of 0.75 [27].

## Discussion

The present study is the first to examine the feasibility and clinical potential of client self-appraisal of risk and protective factors for re-offending in forensic psychiatry. It shows that risk assessment by the client is feasible. Almost without exception, forensic psychiatric outpatients were able to complete the specially developed Client Self-Appraisal based on START.

With respect to the clinical value of risk assessment by the client, analysis at the group level might give the wrong impression that client risk assessment does not add much to risk assessment by the clinician. Clients and clinicians as a group frequently choose the same client characteristics as key risk and protective factors. However, on the individual level agreement between individual clients and their own case manager was only slightly better than chance, both in the choice of key risk and protective factors and in the assessment of client functioning on these factors. This means that in most treatment plan evaluations, the client and case manager come to the evaluation with markedly different ideas about what the important risk and protective factors of the client are, and how the client is doing on these factors. Such divergent views between the client and clinician form the point of departure for the SDM model of treatment [6]. SDM suggests that the client and clinician discuss their differing views and strive for agreement on a treatment plan that does justice to both perspectives. The RNR and GLM models [3, 5], on the other hand, both build on one of the competing views to select treatment targets, with the risk of discontent and disinterest in treatment on the part of the client. Advancing client motivation for treatment is crucial in outpatient forensic psychiatry, where many-in our sample even more than half-of the clients do not (or no longer) have a legal order to enforce treatment. Not addressing the client’s self-perceived treatment needs may then become a fundamental mistake in treatment strategy.

Apart from advancing treatment motivation, addressing the client’s self-perceived risk and protective factors for re-offending may also be expected to improve the effectiveness of treatment. In the present study client risk assessment was found to contribute to the prediction of violent or criminal behaviour by the client in the subsequent months. According to the RNR model, forensic psychiatric treatment should focus on factors which have been shown to be predictive of re-offending by the client; denoted as the client’s criminogenic needs for treatment [3]. The present study shows that key risk and protective factors identified by the client belong to these criminogenic needs and should be addressed to reduce the client’s risk for re-offending.

The independent predictive value of client risk assessment also shows that self-report can provide valid information on the risk and protective factors of forensic psychiatric clients. Several authors warn for the effects of social desirability and ‘impression management’ in self-report assessment of such undesirable characteristics as hostility, anger, and aggressiveness in forensic populations [28, 29]. However, Mills and Kroner [30] conclude from a series of studies in offender populations [30–32], that self-report measures of antisocial traits have predictive validity for criminal offences, despite the influence of social desirability. This is confirmed by a meta-analysis of studies comparing self-report and risk-appraisal by others [33], which showed that self-report inventories designed for criminal and antisocial populations perform at par with the best risk-assessment instruments in predicting criminal justice outcomes. Furthermore, incremental validity analyses conducted as part of this meta-analysis revealed that self-report measures account for as much unique variance in crime-relevant outcomes as risk appraisal by others, and that these methodologies can therefore effectively supplement one another. The results of the present study corroborate these conclusions for risk assessment by forensic psychiatric outpatients. Client self-assessed risk and protective factors proved to have independent predictive validity for violent or criminal behaviour by the client, and the best prediction was obtained when these client self-assessments were combined with the final risk estimate of the case manager after structured risk assessment. The incremental predictive value of client self-perceived violence risk to risk assessment by the case manager was confirmed recently in a study in which clients were asked to express their self-perceived risk for violence by a single global rating [34]. The present study extends this finding to client self-assessment on a comprehensive set of risk and protective factors, as defined by the START. Together these findings underscore the conclusion of the above mentioned meta-analysis [33], that it is time now to work on developing schemes that integrate self-report and risk appraisal by others, and to determine precisely how these two methods can be effectively synthesized to facilitate treatment. We developed one way to integrate risk assessment by the client and case manager (outlined above under ‘Study setting and participants’), and studied the effect of this protocol in the RACE-study.

In the RACE study we tested whether shared care planning based on risk assessment by both the client and case manager reduces the incidence of violent or criminal behaviour in outpatient forensic psychiatry [22]. No effect of the intervention was found. Several explanations for this finding were considered (see [22] for an extensive discussion), but inclusion of client self-appraisal of risk and protective factors and care planning along SDM lines were considered to be strengths of the study [22]. Both elements can get clients more involved in their treatment, and this is of particular importance for forensic psychiatry, where clients often come into treatment under formal or informal coercion. Furthermore, the present study shows that by these methods clients can help their clinician acquire a more comprehensive view of their treatment needs.

### Study limitations

Our study has several limitations. First, 35 % of the clients of the experimental arm of the RACE-study did not receive the intervention, which included the Client Self-Appraisal based on START [22]. The finding that 98 % of the clients who received the intervention could answer the CSA, should therefore be interpreted with some caution. However, there are no indications that inability to complete the CSA played any role in the case manager’s decision to refrain from implementing the intervention [22].

Second, the CSA does not ask clients to assess their functioning on all risk and protective factors of the START, but only on those which the client selected as key factors. For research purposes assessment on all factors may be desirable, for example to compare the predictive value of the sum of all factors to that of the key factors selected by the client. Part 2 of the CSA could then be extended to cover all factors. But for clinical practice instruments should be as concise as possible, and the primary objective for using the CSA will be to discuss with the client what he considers his most important risk and protective factors, and how he thinks he is doing on these factors. Assessment of client functioning on these key factors will then be sufficient. Moreover, the mean scores on these client-selected key risk and protective factors proved to be predictive of client behaviour, as opposed to the case manager’s scoring on their selection of key factors of the START.

Third, nesting of clients within case managers (147 clients of 19 case managers) and of repeated assessments within clients (up to 3 assessment per client) may have affected the results of the prediction analysis. Unfortunately, the limited numbers of available observations (*n* = 175) precluded any meaningful analysis of variability in predictive utility of measures over case managers and repeat assessments.

Finally, the outcome of violent or criminal behaviour in the prediction analyses consisted of incidents recorded by the case manager in the client’s case file. However, case managers may not be aware of all incidents of violent or criminal behaviour of their clients, or they may have received biased information on incidents (e.g. from the client, relatives, police, or probation officer), and not all recorded incidents may meet the criteria of a criminal offence. This may be true, and a check of the predictive validity of the CSA against criminal records would provide valuable additional evidence, but the incidents recorded in the client’s file do constitute the information that is available to case managers in clinical practice, and form the basis on which they monitor treatment progress. These incidents therefore constitute clinically relevant outcomes for the evaluation of the predictive validity of risk assessment in outpatient forensic psychiatry.

## Conclusions

This study demonstrates that the Client Self-Appraisal based on START can be administered in routine outpatient forensic psychiatric care. The instrument enables a comparison between the views of the client and case manager concerning problem behaviour and treatment needs of the client. In general, these views may be expected to differ considerably. It was shown that future violent or criminal behaviour by the client is best predicted by a combination of risk assessment by the client and case manager. Together they know best what should be addressed in treatment to reduce the risk of re-offending. Shared decision making in care planning therefore seems indicated. The Client Self-Appraisal based on START can be a useful tool to facilitate the provision of such forensic psychiatry *with* the client [35].

## Additional file

Additional file 1:
**Client Self-Appraisal based on START (CSA).** New assessment instrument.
