# Supplementary material for: Risk assessment by client and case manager for shared decision making in outpatient forensic psychiatry
Source: BMC Psychiatry. 2015 May 27;15:120. doi: 10.1186/s12888-015-0500-3 (PMC4443540; doi:10.1186/s12888-015-0500-3)
Supplement: Additional file 1: — Client Self-Appraisal based on START (CSA). New assessment instrument. [file 12888_2015_500_MOESM1_ESM.pdf]

Client Self-Appraisal based on START (CSA)

Client: ___________________________ **Date**: ___________________________

PART 1

Vulnerability points

- ***Vulnerability points*** are things in yourself or in your life which can put you at risk for not doing well, and can bring you into contact with the police and legal system again.
- Please indicate for the points below whether they are a vulnerability point of yours, or not
- Mark your answer in the squares. The use of the circles is explained later.

	Point	Vulnerability point? Yes	Somewhat	No	Major points (3 or more)
**1.**	**<u>Difficulty making contact with others</u>**<br>Being withdrawn. Quiet. Finding it difficult to have a conversation with strangers.	☐	☐	☐	○
**2.**	**<u>Trouble building relationships</u>**<br>With friends, a partner, care providers. Difficulty getting along with others, or to feel close to others.	☐	☐	☐	○
**3**	**<u>Problems with work or education</u>**<br>Lacking interest. Finding it difficult to do it right.	☐	☐	☐	○
**4.**	**<u>Lacking leisure activities</u>**<br>Having few hobbies or interests. Being bored, or just hanging around.	☐	☐	☐	○
**5.**	**<u>Difficulty with self-care</u>**<br>Trouble to keep the house clean, prepare a meal, wash your clothes, take a shower regularly, lead a healthy life.	☐	☐	☐	○
**6.**	**<u>Problems with thinking clearly</u>**<br>Difficulty concentrating. Hearing voices, or having intrusive thoughts. Being confused. Having memory problems.	☐	☐	☐	○
**7.**	**<u>Experiencing mood problems</u>**<br>Feeling unpleasant. Depressed. Irritable. Angry.	☐	☐	☐	○
**8.**	**<u>Problems with alcohol or drugs</u>**<br>Drinking too much. Using drugs. Usage that affects your life.	☐	☐	☐	○
**9.**	**<u>Lack of self-control</u>**<br>Doing before thinking. Ignoring the consequences of what you are doing.	☐	☐	☐	○
**10.**	**<u>Susceptible to bad influences</u>**<br>Joining in with friends when you know it will get you into trouble. Finding it hard to resist others.	☐	☐	☐	○
**11.**	**<u>Lacking support of people around you</u>**<br>Having only yourself to rely on. Or rejecting support of others.	☐	☐	☐	○

	Point	Vulnerability point? Yes	Somewhat	No	Major points (3 or more)
**12.**	**Problems with money or housing**<br>Large debts. Lacking money for rent, food, electricity. Having poor or unstable housing. Refusing help in managing your finances.	☐	☐	☐	○
**13.**	**Lacking concern for others**<br>Lying. Difficulty understanding why others feel sad. What you want is more important than what other people want.	☐	☐	☐	○
**14.**	**Stopping or misusing medication**<br>Not taking prescribed medication. Or using it in a different way than prescribed. Doubting the need for medication.	☐	☐	☐	○
**15.**	**Difficulty to keep to the rules**<br>Finding it hard to see the point of rules, or to obey them. Resisting checks, such as a drug test.	☐	☐	☐	○
**16.**	**Behaving unpleasantly towards others**<br>Bullying or frightening others. Insulting others. Stealing or destroying property. Sexually harassing others.	☐	☐	☐	○
**17.**	**Being unsure about your strengths and weaknesses**<br>Unclear to you why you get into trouble sometimes. It always catches you by surprise when things go wrong with you.	☐	☐	☐	○
**18.**	**Missing a goal and plans**<br>Being unsure about what you really want. Having plans you know are unrealistic, or bad for others.	☐	☐	☐	○
**19.**	**Difficulty handling problems**<br>Problems overwhelm you. Make you anxious. Are often too much for you. Coping with changes is difficult for you.	☐	☐	☐	○
**20.**	**Difficulty cooperating with your treatment**<br>Doubting whether treatment is necessary. Pretending to cooperate. Others should change, not you.	☐	☐	☐	○
**21.**	**Problems dealing with sexuality**<br>Missing a sexual partner. Having disturbing or inadmissible sexual urges, fantasies, or behaviors. Unsafe sex. Being addicted to sex.	☐	☐	☐	○
**Other things which can put you at risk of not doing well**					
....		☐	☐	☐	○
....		☐	☐	☐	○

Now turn to the circles at the right hand side. Please mark the 3, or more, most important points for you, which can put you at risk of not doing well.

Protective points

- ***Protective points*** are things in yourself or in your life which can help things go well with you, and keep you out of contact with the police and legal system.
- Please indicate for the points below whether they are a protective point of yours, or not
- Use the squares to mark your answer. We will come back to the use of the circles later again.

	Point	Protective point?			Major points (3 or more)
		**Yes**	**Somewhat**	**No**	
**1.**	**Able to make contact with others**<br>Able to strike up a conversation with somebody. Participating in group activities. Enjoying contact with others.	☐	☐	☐	○
**2.**	**Able to build relationships**<br>With friends, a partner, care providers. Getting along with others. Feeling close to others.	☐	☐	☐	○
**3**	**Enjoying your work or education**<br>Having work or getting an education. Being able to do your work or school well. Showing initiative in the classroom or your job.	☐	☐	☐	○
**4.**	**Having leisure activities**<br>Having hobbies. Being able to entertain yourself.	☐	☐	☐	○
**5.**	**Adequate self-care**<br>Keeping your house clean. Preparing meals. Washing your clothes. Taking a shower regularly. Leading a healthy life.	☐	☐	☐	○
**6.**	**Thinking clearly**<br>Able to focus your mind on something, and to think things through clearly.	☐	☐	☐	○
**7.**	**Having a stable and positive mood**<br>Feeling pleasant. Calm. Hopeful. Having a sense of humor.	☐	☐	☐	○
**8.**	**Having control over your alcohol and drugs usage**<br>Keeping off drugs. Drinking in moderation. Accepting treatment for this, if necessary.	☐	☐	☐	○
**9.**	**Self-control**<br>Able to control yourself. To have a grip on yourself. To think first and act later. Able to deal with disappointments.	☐	☐	☐	○
**10.**	**Withstanding bad influences**<br>Resisting friends who want you to do the wrong things. Having friends who help to do the right things. Making up your own mind.	☐	☐	☐	○
**11.**	**Having support of people around you**<br>Having people who support you (family, friends or care providers). Who mean well with you.	☐	☐	☐	○

	Point	Protective point? Yes	Somewhat	No	Major points (3 or more)
12.	**Having sufficient money and adequate housing**<br>Managing with your money. Being without debts. Having adequate housing. Accepting help in administering your money, if needed.	☐	☐	☐	○
13.	**Respecting others**<br>Being open and honest to others. Showing concern for others. Seeing others as equals.	☐	☐	☐	○
14.	**Making appropriate use of medication**<br>Using prescribed medication, and in the right way. Understanding the need for medication.	☐	☐	☐	○
15.	**Obeying rules**<br>Understanding why rules are necessary. Cooperating with checks, such as a drug test.	☐	☐	☐	○
16.	**Behaving pleasantly towards others**<br>Respecting other people's property. Being considerate about others and their safety. Arriving on time for appointments.	☐	☐	☐	○
17.	**Knowing your strengths and weaknesses**<br>Knowing why you get into trouble sometimes. Recognizing when you are no longer doing well. Aware of your problem areas.	☐	☐	☐	○
18.	**Having a goal and plans**<br>Having something to strive for, which is not at the expense of others. Having realistic plans.	☐	☐	☐	○
19.	**Able to handle difficulties**<br>Being able to solve your problems. To deal with stress and changes. To ask for help, if needed. To look on the bright side.	☐	☐	☐	○
20.	**Cooperating with your treatment**<br>Being committed to your treatment. Wanting to succeed. Cooperating, because you want it yourself.	☐	☐	☐	○
21.	**Managing sexuality adequately**<br>Having a good sexual relationship. Having control over your sexual urges, fantasies and behavior. Safe sex for both.	☐	☐	☐	○
	**Other points which can help things go well with you**				
	....	☐	☐	☐	○
	....	☐	☐	☐	○

Now turn to the circles at the right hand side. Please mark the 3, or more, most important points for you, which can make things go well with you.

PART 2

You just selected your major vulnerability and protective points. These are the points for which you marked the circle on the right side of the page.

Please copy your choices to the tables below. Write the numbers of your major vulnerability points in the top table, and the numbers of your major protective points in the bottom table.

Now for each point rate how you are doing on that point at the moment. Do this by circling the appropriate number out of the range from 0 till 10. The meaning of the numbers is:

0	=	Could not be worse
6	=	Just sufficient
10	=	Could not be better

**Major vulnerability point**	**How are you doing on this point at the moment?**										
Number	**Could not be worse**						**Just Sufficient**				**Could not be better**
	**0**	**1**	**2**	**3**	**4**	**5**	**6**	**7**	**8**	**9**	**10**
	**0**	**1**	**2**	**3**	**4**	**5**	**6**	**7**	**8**	**9**	**10**
	**0**	**1**	**2**	**3**	**4**	**5**	**6**	**7**	**8**	**9**	**10**
	**0**	**1**	**2**	**3**	**4**	**5**	**6**	**7**	**8**	**9**	**10**
	**0**	**1**	**2**	**3**	**4**	**5**	**6**	**7**	**8**	**9**	**10**

**Major protective point**	**How are you doing on this point at the moment?**										
Number	**Could not be worse**						**Just Sufficient**				**Could not be better**
	**0**	**1**	**2**	**3**	**4**	**5**	**6**	**7**	**8**	**9**	**10**
	**0**	**1**	**2**	**3**	**4**	**5**	**6**	**7**	**8**	**9**	**10**
	**0**	**1**	**2**	**3**	**4**	**5**	**6**	**7**	**8**	**9**	**10**
	**0**	**1**	**2**	**3**	**4**	**5**	**6**	**7**	**8**	**9**	**10**
	**0**	**1**	**2**	**3**	**4**	**5**	**6**	**7**	**8**	**9**	**10**

© Short-Term Assessment of Risk and Treatability (START): BC Mental Health & Addiction Services, Coquitlam, British Columbia, Canada and St. Joseph's Healthcare Hamilton, Ontario, Canada, 2004 and 2009
© Client Self-Appraisal based on START: RGOc, University Medical Center Groningen, Groningen, the Netherlands, 2011
